# Personal

**Published:** 1891-01

**Authors:** 


### Personal.

Colonel Charles Sutherland, Surgeon U. S. Army, has been appointed Surgeon-General, with the rank of Brigadier-General, vice Jedediah H. Baxter, deceased. General Sutherland entered the military service August 5, 1852, as Assistant Surgeon, having been appointed from Pennsylvania. He has been in continuous service ever since, and brings to his new position as Surgeon-General a rare equipment in experience and personal fitness.

Dr. Carl Weidner, of Louisville, Demonstrator of Bacteriology in the Kentucky School of Medicine, has been sent to Berlin by Dr. J. B. Marvin, Professor of Practice in the same school. Dr. Weidner is Dr. Marvin's assistant, a successful practitioner, and an expert microscopist. He goes to Berlin to investigate practically the Koch method of the treatment of tuberculosis, and will remain some time.

Dr. L. Bradley Dorr has been appointed Assistant in Chemistry at the Medical Department of the Niagara University. Dr. Dorr has enjoyed the excellent training offered by the Johns Hopkins University. The Niagara University is fortunate in securing so able a man to fill the place left vacant by Mr. Jessel's resignation.

Dr. J. C. Sexton, of Rushville, Ind., is the author of some very bright and sensible sayings in his presidential address before the Union District Medical Society, at Connersville, October 23, 1890, and which is published in the December number of the *Indiana Medical Journal.*

Dr. T. Haven Ross announces to the profession that he has made a special study of Orthopedic Surgery for several years, and that he has opened a down-town office (temporarily) at 360 Main street, Room 8, for the practice of that specialty.

Dr. Gustav A. Pohl has removed to 96 Lemon street, above Virginia street, Buffalo, N. Y. Office hours: 8.30 a. m., 1 and 7 p. m. Sundays, 9 a. m.

